# Research trends and hotspots of exercise for Alzheimer’s disease: A bibliometric analysis

**DOI:** 10.3389/fnagi.2022.984705

**Published:** 2022-09-07

**Authors:** Binglin Chen, Yujie Fu, Ge Song, Weiquan Zhong, Jiabao Guo

**Affiliations:** ^1^The Second Clinical Medical College, Xuzhou Medical University, Xuzhou, China; ^2^Department of Rehabilitation Medicine, Ruijin Hospital, Shanghai Jiao Tong University School of Medicine, Shanghai, China

**Keywords:** exercise, CiteSpace, Alzheimer’s disease, visualized analysis, bibliometrics

## Abstract

**Objective:**

Alzheimer’s disease (AD) is a socially significant neurodegenerative disorder among the elderly worldwide. An increasing number of studies have revealed that as a non-pharmacological intervention, exercise can prevent and treat AD. However, information regarding the research status of this field remains minimal. Therefore, this study aimed to analyze trends and topics in exercise and AD research by using a bibliometric method.

**Methods:**

We systematically searched the Web of Science Core Collection for published papers on exercise and AD. The retrieved data regarding institutions, journals, countries, authors, journal distribution, and keywords were analyzed using CiteSpace software. Meanwhile, the co-occurrence of keywords was constructed.

**Results:**

A total of 1,104 papers were ultimately included in accordance with our specified inclusion criteria. The data showed that the number of published papers on exercise and AD is increasing each year, with papers published in 64 countries/regions and 396 academic journals. The Journal of Alzheimer’s Disease published the most papers (73 publications). Journals are concentrated in the fields of neuroscience and geriatrics gerontology. The University of Kansas and the United States are the major institution and country, respectively. The cited keywords show that oxidative stress, amyloid beta, and physical exercise are the research hotspots in recent years. After analysis, the neuroprotective effect of exercise was identified as the development trend in this field.

**Conclusions:**

Based on a bibliometric analysis, the number of publications on exercise and AD has been increasing rapidly, especially in the past 10 years. “Amyloid beta,” “oxidative stress,” and “exercise program” trigger the most interest among researchers in this field. The study of exercise program and mechanism of exercise in AD is still the focus of future research.

## Introduction

Alzheimer’s disease (AD) is an age-related neurodegenerative disorder caused by damage to brain neurons (Alzheimer’s Association, 2022). Hallmark pathological changes include neuritic extracellular amyloid plaque and neurofibrillary tangles (Barthelemy et al., 2020). AD is characterized by cognitive decline and behavioral changes with memory, language, and thinking problems as the initial symptoms (Dubois et al., 2016, 2018). Progressive symptoms will continue to affect activities of daily living. The rate in which AD progresses varies from person to person. An estimated 55 million people living with dementia worldwide were reported by Alzheimer’s Disease International in 2021, and patients with AD are expected to increase to 78 million by 2030 (Alzheimer’s Disease International, 2021). AD accounts for the largest proportion of patients with dementia, and the proportion of those over 60 years old is about 65% (Jia et al., 2020). AD is a fatal illness, and the average survival time of AD is reported to vary from 4 to 8 years for patients aged 65 years and older (Larson et al., 2004). Globally, given the large population of patients with AD and the harmfulness of this disease, AD has become a considerable health and economic burden, calling for more effective measures to control this disease.

To date, AD is treatable but not curable. Current interventions are mostly aimed at slowing down the progression of AD, reducing its symptoms, and improving quality of life. Exercise is considered an important lifestyle modification that can help delay the beginning of cognitive deterioration and improve the quality of life of patients with AD (Lautenschlager et al., 2008; Petersen et al., 2018). Numerous studies have found that aerobic exercise, resistance exercise, and cognitive–physical exercise can help patients with AD in many aspects, such as improving cardiovascular fitness, attenuating neuroinflammation, and supporting the brain clearance of Aß peptides (Karssemeijer et al., 2017; Morris et al., 2017; Sun et al., 2018; Ribaric, 2022). Given the increase in the number of publications about the use of exercise in treating AD, identifying research trends and hotspots is highly significant. However, a quantitative analysis of this research theme has not yet been conducted.

Bibliometric analysis is a scientific method for constructing a co-occurrence network of research themes by using quantitative statistics (Hicks et al., 2015). The software tool, CiteSpace, is used to show a visual map of bibliometric results, such as journals, authors, institutes, keywords, citations, popular topics, and frontiers. Some reviews on the use of exercise to treat AD with different emphases have been published; however, a comprehensive and visualized analysis remains lacking (Karssemeijer et al., 2017; Ribaric, 2022). Therefore, we conduct a bibliometric analysis of exercise and AD research to reveal the dynamic development in this field. This study helps provide a comprehensive understanding of this topic and guide future research direction.

## Methods

### Search strategy

**FIGURE 1:**
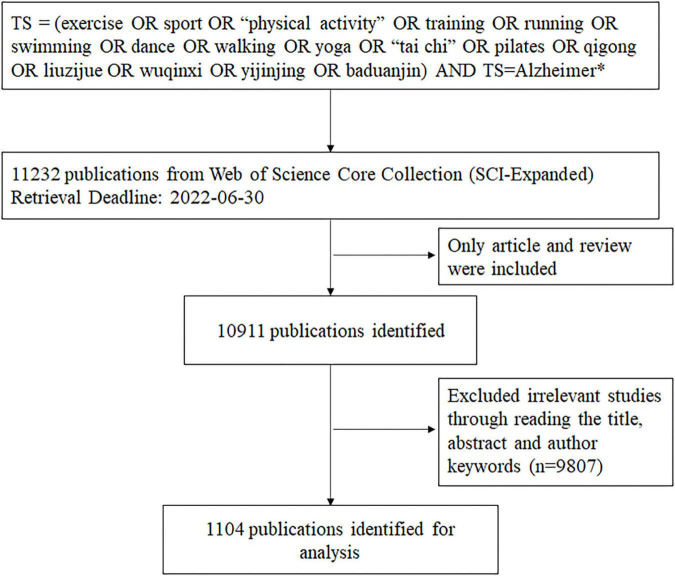
Flow diagram of the publications screening process.

The data used in this study were collected from the Science Citation Index Expanded (SCI-E) of the Web of Science Core Collection database. The search strategy was as follows: TS = (exercise OR sport OR “physical activity” OR training OR running OR swimming OR dance OR walking OR yoga OR “tai chi” OR pilates OR qigong OR liuzijue OR wuqinxi OR yijinjing OR baduanjin) AND TS = Alzheimer*. The search strategy identified papers with these words mentioned in their title, abstract, author keywords, or keywords plus. Only articles and reviews were included as document types. The time span was from inception to June 30, 2022. All papers from the search were preliminarily included, and we screened all the papers by reading the title, abstract and author keywords, and excluded irrelevant literature (e.g., “computer running,” “speech training,” and “common training library”). Discrepancies were observed via discussion. Figure 1 shows the flow diagram of the publications screening process.

### Analytical tool

CiteSpace (Chaomei, 2006) is a visual analysis application developed by Dr. Chen Chaomei of Drexel University. It is based on theory of citation analysis, which has been applied by many scholars worldwide. CiteSpace is well recognized for transforming quantitative literature data into visual maps and networks to provide key information, including research trends, popular topics, and distribution of countries. Cluster and time-zone views are included in CiteSpace’s visualization. Visual networks have been confirmed significant in research trends and key points. We can see different nodes and links in various CiteSpace visualization knowledge maps. Nodes represent different key points, countries, institutions, and journals. The larger the nodes, the greater number of occurrences or citations in this field. Different colors represent various years. A relatively early time is represented by cold-hued nodes, while a relatively late time is represented by warm-hued nodes. The centrality of a node indicates the importance of a node’s status in a network. In CiteSpace, a node with a purple ring is considered a pivotal point with high centrality (Chen, 2017). Microsoft Excel (2019) was used to generate a graph of the trends in annual publications and citations.

## Results

### Publication trends

**FIGURE 2:**
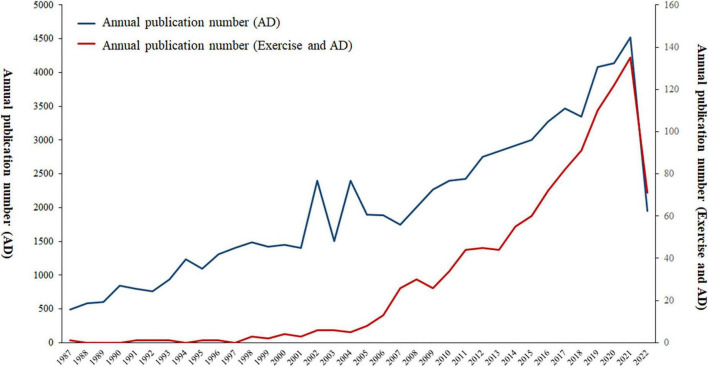
Annual number of publications on AD, exercise and AD from 1987 to 2022.

A total of 1,104 publications met our inclusion criteria. Researchers are paying more and more attention to AD each year. Consistent with this, despite some minor fluctuations but with an overall upward trend for the number of studies on exercise intervention in AD (Figure 2). The volume of published literature can be broadly divided into three periods: 1987–2000, 2000–2006, and 2006–2021. The number of publications in the first period was low, while the number of publications in the second and third periods increased significantly. The maximum number of relevant publications (*n* = 135) was reached in 2021. A considerable increase occurred in the second period, while the largest increase was recorded in the third period, with a number of 122 additional pieces of literature from 2006 to 2020. The citations of literature have largely increased every year, with the most significant increase occurring from 2016 to 2021. The large numbers of publications and literature citations in recent years have indicated the attention given by scholars to this area.

### Analysis of countries/regions and institutions

The literature came from 64 countries/regions. In accordance with Table 1, among all the countries/regions where literature was published, the top 3 in terms of number of publications are as follows: the United States (424 publications), China (137 publications), and Brazil (86 publications). Among these countries/regions, the top three in terms of citations to literature are the United States, Australia, and Canada. Meanwhile, the countries with the highest average citations per item are Australia, Germany, and the United States in that order. The top three countries in terms of the *h*-index are the United States, Australia, and Germany. The United States dominates the field, with the highest number of publications, citations, centrality, and *h*-index. Although China and Brazil have more publications, they have fewer citations and their academic influence is more limited.

**TABLE 1:** Top 10 most productive countries/regions.

Country/Region	Publications	Citations	Average citations per item	Centrality	H-index
United States	424	25318	59.71	0.61	80
China	137	2561	18.69	0.08	27
Brazil	86	2280	26.51	0.01	26
Australia	77	4780	62.08	0.11	32
Canada	76	4061	53.43	0.04	25
United Kingdom	65	3055	47.00	0.08	30
Spain	63	1891	30.02	0.08	25
Germany	60	3680	61.33	0.04	30
Italy	49	1802	36.78	0.04	23
Japan	47	2509	53.38	0.06	23

**FIGURE 3:**
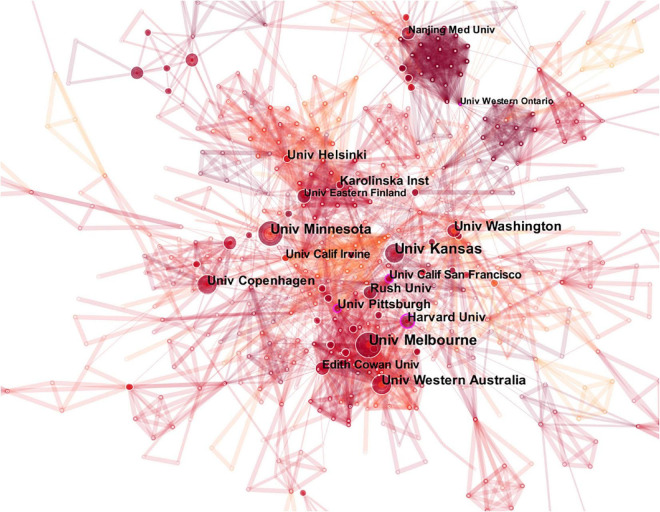
The map of co-institutions. The nodes in the map represent co-institutions, and lines between the nodes represent co-citation relationships. The purple ring represents centrality.

Table 2 lists the 10 institutions with the highest number of publications. The most productive institution is the University of Kansas (31 publications), and the University of Melbourne (28 publications) has the second highest number of publications, followed by the University of Minnesota (27 publications). Figure 3 illustrates the collaborative relationships among different institutions.

**TABLE 2:** Top 10 most productive institutions.

Rank	Institution	Publications	Rank	Institution	Centrality
1	University of Kansas	31	1	University of Pittsburgh	0.14
2	University of Melbourne	28	2	University of Western Ontario	0.14
3	University of Minnesota	27	3	University of California San Francisco	0.12
4	University of Washington	22	4	Harvard University	0.11
5	University of Western Australia	21	5	University of Washington	0.1
6	University of Pittsburgh	18	6	Autonomous University of Barcelona	0.08
7	Harvard University	18	7	University of Melbourne	0.06
8	University of Copenhagen	17	8	Karolinska Institutet	0.06
9	Rush University	16	9	Edith Cowan University	0.06
10	Karolinska Institutet	16	10	University of Eastern Finland	0.06

Links between the University of Melbourne, Harvard University, University of Pittsburgh, University of California San Francisco and other institutions indicate close collaboration. The centrality of the University of Pittsburgh, the University of Western Ontario, the University of California San Francisco and Harvard University is greater than 0.1, demonstrating a wide range of academic influence.

### Analysis of authors

**FIGURE 4:**
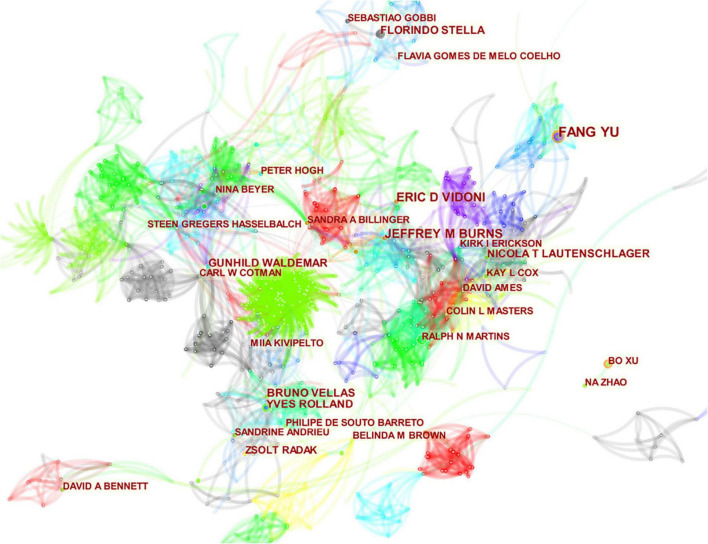
The map of co-authors. The nodes in the map represent co-authors, and lines between the nodes represent co-citation relationships. The purple ring represents centrality.

The papers were contributed by 5,267 authors. The top 10 authors in terms of number of publications are presented in Table 3. The top three most frequently cited authors were Cotman CW (2,387 citations), Lautenschlager NT (1,488 citations), and Cox KL (1,322 citations). Cotman CW had a significantly higher number of citations than the other authors. He also had the highest number of citations per item and the highest *h*-index, endowing him with greater academic influence. As shown in Figure 4, the field has formed a relatively large number of research teams, with many highly productive authors among them. The major research teams are those of Yu F, Cotman CW, Burns JM, Zhang L, and Hasselbalch SG.

**TABLE 3:** Top 10 most productive authors.

Author	Publications	Citations	Average citations per item	H-index
Yu, Fang	24	321	13.38	11
Burns, Jeffrey M.	22	797	36.23	13
Vidoni, Eric D.	18	281	15.61	9
Lautenschlager, Nicola T.	14	1488	106.29	11
Hasselbalch, Steen Gregers	14	462	33	12
Cotman, Carl W.	13	2387	183.62	12
Stella, Florindo	12	397	33.08	10
Strandberg, Timo	12	460	38.33	9
Cox, Kay L.	12	1322	110.17	9
Rolland, Yves	12	1048	87.33	9

### Analysis of journals and categories

All the retrieved publications were published in 396 different journals. Table 4 lists the top 10 journals that are publishing in this area. The top five journals in terms of number of publications were as follows: Journal of Alzheimer’s sisease (73 publications), Frontiers in Aging Neuroscience (34 publications), Behavioral Brain Research (26 publications), Current Alzheimer Research (21 publications), and International Journal of Molecular Sciences (21 publications). Among the top 10 journals, the Journal of Alzheimer’s Disease was the most cited (2,435 citations), while the Journal of the Neurology had the highest average number of citations per term (128.56). Publications from the top 10 journals were primarily published in Neurosciences (409 publications) and Geriatrics and Gerontology (268 publications). The top ten web of science categories in this field also include: Clinical Neurology (177 publications), Gerontology (93 publications), Psychiatry (76 publications), Sport Sciences (76 publications), Biochemistry Molecular Biology (62 publications), Medicine General Internal (54 publications), Behavioral Sciences (50 publications), and Medicine Research Experimental (44 publications).

**TABLE 4:** Top 10 most productive journals.

Journals	Publications	Citations	Average per item citations	2021 IF
Journal of Alzheimer’s disease	73	2435	33.34	4.160
Frontiers in Aging Neuroscience	34	659	19.38	5.702
Behavioral Brain Research	26	962	36.92	3.352
Current Alzheimer Research	21	425	20.24	3.040
International Journal of Molecular Sciences	21	189	9.00	6.208
American Journal of Alzheimer’s Disease and Other Dementias	18	380	21.11	2.632
Journal of the American Geriatrics Society	16	1260	78.75	7.538
Neurology	16	2058	128.56	11.800
Neurobiology of Aging	15	823	54.87	5.133
Alzheimer and Dementia	14	673	48.07	16.655

### Analysis of the top 10 most cited papers

Table 5 lists the top 10 papers based on the number of total citations and average per year on exercise and AD research, which focused on the research themes of cognitive function, exercise plus behavioral management, cognitive training, and brain plasticity. The most cited papers was “An active and socially integrated lifestyle in late life might protect against dementia” by Fratiglioni L and published in the Lancet Neurology in 2004. The top-ranked papers were those published earlier, indicating that they have been consistently cited and have high reference value. Based on average per year of citations on exercise and Alzheimer’s research, “Exercise-linked FNDC5/irisin rescues synaptic plasticity and memory defects in Alzheimer’s models” by Lourenco MV in 2019 (68.5 citations per year), “Combined adult neurogenesis and BDNF mimic exercise effects on cognition in an Alzheimer’s mouse model” by Choi SH in 2018 (59 citations per year), and “Physical Activity, Cognition, and Brain Outcomes: A Review of the 2018 Physical Activity Guidelines” by Erickson KI in 2019 (56.5 citations per year) were relatively new in the field, focusing on the study of exercise for brain plasticity.

**TABLE 5:** The top 10 papers based on the number of citations on exercise and AD research.

Title	Journal	Year	Citations
**Total**			
An active and socially integrated lifestyle in late life might protect against dementia	Lancet Neurology	2004	1217
Leisure activities and the risk of dementia in the elderly	New England Journal of Medicine	2003	1110
Effect of physical activity on cognitive function in older adults at risk for Alzheimer disease: a randomized trial	Journal of the American Medical Association	2008	1032
Physical activity and risk of cognitive impairment and dementia in elderly persons	*Archives of Neurology*	2001	952
Exercise is associated with reduced risk for incident dementia among persons 65 years of age and older	Annals of Internal Medicine	2006	873
Risk factors for Alzheimer’s disease: a prospective analysis from the Canadian Study of Health and Aging	American Journal of Epidemiology	2002	862
The effects of exercise training on elderly persons with cognitive impairment and dementia: A meta-analysis	Archives of Physical Medicine and Rehabilitation	2004	799
Effects of aerobic exercise on mild cognitive impairment: a controlled trial	*Archives of Neurology*	2010	718
Leisure-time physical activity at midlife and the risk of dementia and Alzheimer’s disease	Lancet Neurology	2005	667
Incidence and risk factors of vascular dementia and Alzheimer’s disease in a defined elderly Japanese population: the Hisayama study	Neurology	2013	643
**Average per year**			
Effect of physical activity on cognitive function in older adults at risk for Alzheimer disease: a randomized trial	Journal of the American Medical Association	2008	68.8
Exercise-linked FNDC5/irisin rescues synaptic plasticity and memory defects in Alzheimer’s models	Nature Medicine	2019	68.5
Long-term health benefits of physical activity: a systematic review of longitudinal studies	BMC Public Health	2013	64.3
An active and socially integrated lifestyle in late life might protect against dementia	Lancet Neurology	2004	64.05
Diagnosis and management of dementia: review	Journal of the American Medical Association	2019	61.75
Combined adult neurogenesis and BDNF mimic exercise effects on cognition in an Alzheimer’s mouse model	Science	2018	59
Physical activity, cognition, and brain outcomes: a review of the 2018 physical activity guidelines	Medicine and Science in Sports and Exercise	2019	56.5
Leisure activities and the risk of dementia in the elderly	New England Journal of Medicine	2003	55.5
Effects of aerobic exercise on mild cognitive impairment: a controlled trial	*Archives of Neurology*	2010	55.23
Bridging animal and human models of exercise-induced brain plasticity	Trends in Cognitive Sciences	2013	52.2

### Analysis of keywords

**FIGURE 5:**
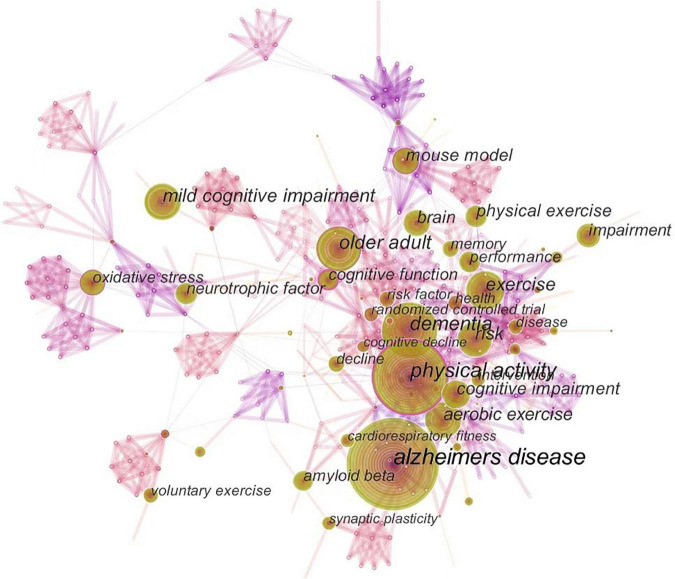
The map of keywords. The circle size and the link illustrated the frequency and relevance of keywords, respectively.

**FIGURE 6:**
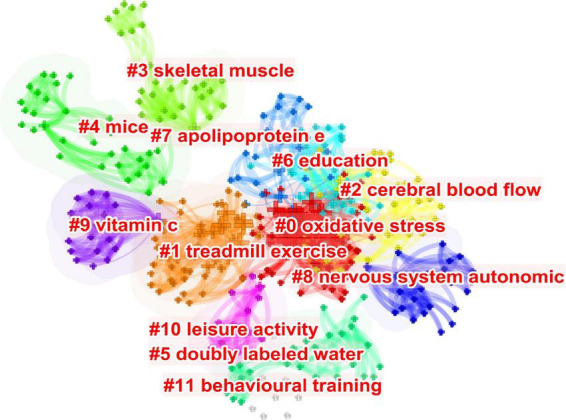
The cluster diagram for the keywords.

**FIGURE 7:**
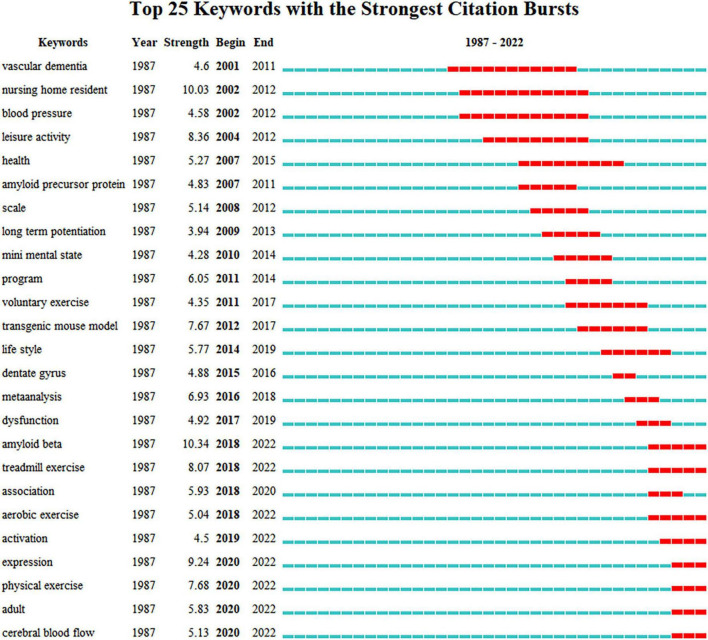
The keywords with the strongest citation bursts of publications. Each blue or red short line represents a year, and a red line stands for a burst detected year.

Figure 5 shows the co-occurrence of keywords, which are highly closely linked to one another. Figure 6 presents the cluster diagram for the keywords. The clusters include oxidative stress, cerebral blood flow, skeletal muscle, mice, doubly labeled water, education, apolipoprotein e, nervous system autonomic, vitamin c, leisure activity, behavioral training. Figure 7 shows the top 25 keywords with the strongest citation bursts since 1987. These keywords are also the research frontiers in the field. The red bars indicate the emergence and duration of research hotspots. The top 25 keywords with the strongest citation bursts began in 2001. These words are mainly concentrated in two categories: program of exercise intervention in AD (e.g., physical exercise, aerobic exercise, treadmill exercise, voluntary exercise, and leisure activity), mechanism of exercise intervention in AD (e.g., amyloid beta, cerebral blood flow, amyloid precursor protein, and long term potentiation). The keywords with the strongest citation bursts are amyloid beta. Combined with the above data and analysis, the major research frontiers in recent years are exercise program, amyloid beta, and oxidative stress.

## Discussion

### Global research trends of exercise on Alzheimer’s disease

This study described the landscape of exercise-based rehabilitation and prevention of AD by analyzing subject categories and the contribution of countries, journals, and authors. A total of 1,104 papers were obtained through the retrieval strategy.

Our study found that research on the relationship between exercise and AD started in 1987, and the number of published papers exhibited an increasing trend every year. The number of published papers in 2021 is 3.97 times that in 2010 and 33.75 times that in 2000. Among all the studies, eight highly cited publications were published in the last 10 years (2013–2021), which might be a period of high-quality development in the field of sports and AD research. The aforementioned findings suggest that exercise and AD are eliciting extensive attention among researchers and have become popular research issues in recent years. This phenomenon may be related to the increase in AD incidence rate with the magnification of the aging society and the rapid development of sports science and rehabilitation medicine (Garcia-Morales et al., 2021; Zong et al., 2022).

By analyzing the journals about exercise and AD, we determined that researchers concentrated in the fields of neuroscience and geriatrics. Among them, the Journal of Alzheimer’s disease (73 publications), Frontiers in Aging Neuroscience (34 publications), Behavioral Brain Research (26 publications), Current Alzheimer Research (21 publications), and International Journal of Molecular Sciences (21 publications) have given the most attention to this field, demonstrating that the research field has focused on neuroscience and gerontology. The top three Web of Science categories are neuroscience, geriatrics gerontology, and clinical neurology. In addition, the top 15 categories include sport science, psychology, behavioral science, biochemistry molecular biology, and rehabilitation. This finding suggests that the exercise-based rehabilitation and prevention of AD are typically multidisciplinary collaborative effort. The etiology of AD is closely related to neuroscience and aging (Atri, 2019; Ribaric, 2022). Meanwhile exercise intervention and prevention belong to the category of sports science and rehabilitation, and their mechanism research involves behavior, cognition, physiology, and biochemistry. Therefore, establishing a multidisciplinary team to conduct exercise intervention and prevention of AD is beneficial.

The quantitative and visual analyses of countries/regions distribution show that the United States is the leading country in the field of exercise-based rehabilitation and prevention of AD, with the highest number of studies (424 publications), citations, centrality, and *h*-index. This result may be due to the internal drive caused by its aging society and the large amount of scientific research capital investment (the United States Department of Health Human Services, National Institutes of Health, and National Institute on Aging are the top three funders for this field, and all of which are United States institutions). Some Asian countries, such as China and Japan, have participated in research on this field and made several achievements. Among them, China has performed well in the number of published papers (137 publications) and the support of funds (National Natural Science Foundation of China, ranked 4th among funding institutions). However, China exhibits no advantages in average per item, citations, and *h*-index, indicating that the quality of research should be further improved. The analysis of the reasons may be as follows: the density and breadth of international cooperation in this field is insufficient for China, and iconic research institutions are lacking. Therefore, high-quality research output is also lacking. This situation may limit the development of research on exercising and AD, because China, the world’s most populous country, is also a country with rapidly increasing aging population, and thus, it urgently needs to make breakthroughs in this field. Therefore, we suggest that European and American research institutions should strengthen cooperation and exchanges with China and institutions to promote the progress of research on the exercise rehabilitation and prevention of AD worldwide.

From the perspectives of author contribution and co-citation, the author’s co-occurrence chart (Figure 4) shows numerous nodes, and the connection between clusters was relatively close, indicating a high number of international researchers in this field. However, the research direction was relatively scattered. As shown in Table 3, Yu F (24 publications, 321 citations, and 11 *h*-indexes), an American researcher from the University of Minnesota, published the largest number of literatures. He began studying the effects of exercise on AD in 2006. This author believes that physical activity and exercise can prevent or relieve the cognitive and functional impairments brought by AD because exercise may improve the pathogenesis of AD and stimulate the brain plasticity of patients (Yu et al., 2006; Gronek et al., 2019; Zong et al., 2022). Another key author named Cotman CW (13 publications, 2,383 citations, and 12 *h*-indexes) had the highest citation on the basis of a high number of papers, showing a good academic influence. He has been conducting research in this area since 1999, focusing on the mechanism of exercise intervention in AD. The author believes that the main way for exercise to improve cognitive function in AD patients is to reduce Aβ deposition (Adlard et al., 2005) and alleviate the neuroinflammation caused by oxidative stress (Parachikova et al., 2008; Ionescu-Tucker and Cotman, 2021). In addition, the authors observed that exercise can restore the hippocampal function in AD patients by enhancing the expression of brain-derived neurotrophic factor (BDNF) and other growth factors that promote neurogenesis, angiogenesis, and synaptic plasticity (Intlekofer and Cotman, 2013; Berchtold et al., 2019).

### Literature review

In accordance with highly cited literature, keyword co-occurrence and explosion analysis cannot only reveal the core contents and research topics of publications in a certain field but also help us learn the current research focus and development trends in this field (Zhang et al., 2022).

#### The effect of exercise on Alzheimer’s disease

According to the keyword burst chart (Figure 7), five burst keywords related to exercise were found: “leisure activity,” “treadmill exercise,” “physical exercise,” “aerobic exercise,” and “voluntary exercise.” Burst keywords “treadmill exercise” and “aerobic exercise,” which appeared in 2018, have continued to appear in 2022. These burst keywords indicated that exercise program is the current research hotspot of the field. The effect of exercise on AD may vary based on the pattern, intensity, and lasting duration of exercise. A meta-analysis has been conducted to compare the effects of different exercise modalities (aerobic exercise, muscle strength training, and combined training) on the function of patients with AD (Lopez-Ortiz et al., 2021). The results of the meta-analysis showed that aerobic exercise can improve the cognitive and physical functions of AD patients, whereas muscle strength training and combined training had no significant effect. The forms of aerobic exercise included in this meta-analysis were cycling, walking, treadmill, and arm ergometry. As for exercise intensity, a medium to high intensity, which was measured by maximum heart rate or heart rate reserve, was usually used. A certain evidence indicates that high-intensity interval training is more beneficial than moderate-continuous exercise training for slowing down the progression of AD. The former can produce higher lactate levels, which elicit larger increases in BDNF, which participates in the neurotrophic signaling pathways of learning and memory function improvement (Boyne et al., 2019; Antunes et al., 2020). However, the study of Jahangiri et al. (2019) supports moderate and regular exercise, and they considered that high-intensive exercise will lead to excessive stress response, which may cause the symptoms of cognitive impairment. In addition, we summarized this meta-analysis and observed that the training time ranged from 30 to 90 min, and training lasted for 2–3 times a week. The total time of intervention ranged from 9 weeks to 9 months. In animal studies, we observed that most of the literature related to physical activity for AD used treadmill exercise (da Costa Daniele et al., 2020). Although numerous works have been conducted on the mechanism research of exercise benefits on AD, no study compared the different effects of various intensities, frequencies, and durations on the mechanism. Further studies evaluating differences in exercise programs are necessary.

#### Potential mechanism of exercise for Alzheimer’s disease

First, we ranked the top 10 references in terms of the number of citations to identify references that may be important in exploring the frontier knowledge base of research. As indicated in Table 3, the paper titled, “Effect of physical activity on cognitive function in older adults at risk for Alzheimer’s disease: a randomized trial,” published by Lautenschlager et al. (2008) in the Journal of the American Medical Association (IF = 56.274) in 2008 was cited 1,033 times. This paper reports the first randomized controlled trial to determine whether physical activity reduces the incidence of cognitive decline among high-risk elderly population. The final results suggested that 6 months of physical activity improved the cognitive performance of AD subjects during the follow-up period of 18 months. This study laid the foundation for subsequent related research. Among the top ten papers cited average per year, “Combined adult neurogenesis and BDNF mimic exercise effects on cognition in an Alzheimer’s mouse model,” was published in Science (IF = 47.728) in 2018 (Choi et al., 2018) and “Exercise-linked FNDC5/irisin rescues synaptic plasticity and memory defects in Alzheimer’s models,” was published in Nature Medicine (IF = 53.44) in 2019 (Lourenco et al., 2019). Both papers focused on and explained the neuroprotective effects of exercise on AD from the perspectives of irisin, neurogenesis, and BDNF. In summary, we determined that the research trend of exercise and AD in recent years has shifted from discussing the influence of exercise on cognitive function to exploring the mechanism of exercise that improves cognitive function in AD patients. The current trend focuses on the neuroprotective effect of exercise.

From the keyword burst chart (Figure 7), the keywords with the highest burst value was “amyloid beta” (bursts strength value 10.03), and the burst has continued since 2018. This finding suggests that exercise clearance “amyloid beta” may be the research front of the mechanism research of exercise intervention in AD. The presence of neurotoxic amyloid plaques, which Aβ forms as a result of a pathological cascade reaction, is considered the gold standard for AD neuropathological diagnosis (Liang et al., 2022). The abnormal accumulation of extracellular Aβ causes evident neurotoxicity, which can induce brain inflammation, mitochondrial dysfunction, oxidative stress induced microglia activation, and other toxic side effects. This condition will exacerbate neuronal loss and promote the development of AD (Kinney et al., 2018). Therefore, removing the excessive accumulation of Aβ is an important train of thought in the treatment of AD. Exercise is considered an effective way to prevent and treat AD; such effectiveness may be related to the capability of exercise to participate in the clearance of the excessive accumulation of Aβ in the brain (Radak et al., 2010; Aczel et al., 2022). Adlard et al. (2005) published the study titled, “voluntary exercise decreases amyloid load in a transgenic model of Alzheimer’s disease,” in the Journal of Neuroscience, and it was possibly the earliest study on the clearance of Aβ by exercise. The authors used TgCRND8 mice as animal models to observe the interaction between 5 months of voluntary exercise and AD cascade. Exercise caused extracellular hippocampus Aβ plaque reduction, and this result was related to the reduction of cortex Aβ 1–40 and Aβ 1–42. The authors believed that this mechanism is mediated by changes in the amyloid precursor protein processing after a short-term exercise. Recent systematic reviews have summarized the effects of involuntary chronic physical exercise on beta-amyloid protein in experimental models of AD. The results from 36 included studies showed that regular physical exercise resulted in positive changes in amyloid precursor protein processing through different signal pathways, thus proving the anti-amyloid effect of exercise (Vasconcelos-Filho et al., 2021). In addition, different studies attempted to clarify the mechanism by which exercise reduces Aβ deposition to protect AD from different perspectives, such as Aβ generation, Aβ transporters crossing the blood–brain barrier, autophagy, degrading enzymes, etc. However, the mechanism by which exercise reduces Aβ deposition has remained unclear until now, therefore becoming the focus of attention of researchers.

Aβ deposition plays a key role in the progression of AD, and other pathological events (including mitochondrial dysfunction, oxidative stress, or neuroinflammation) contribute significantly to its development (Tan et al., 2021). According to the keyword centrality and cluster analysis results (Figures 5, 6), studies related to “oxidative stress” have attracted wide attention. Oxidative stress causes mitochondrial dysfunction, which is associated with the development of AD-related pathology (Yu et al., 2018). In addition, oxidative stress promotes Aβ deposition during the development of AD. The excessive accumulation of Aβ induces oxidative stress of microglia, resulting in chronic neuroinflammation and further aggravating the oxidative stress-induced nerve damage (Liang et al., 2021). Exercise is closely related to the improved antioxidant capacity of the brain and reduced oxidative stress-induced injury (Liang et al., 2021). TgF344-AD rats were used as models to observe the effect of 8 months of exercise pre-training on AD. The results showed that exercise pre-training reduced Aβ deposition and tau hyperphosphorylation, inhibited mitochondrial dynamic imbalance, and significantly inhibited oxidative stress and neuroinflammation in AD rats (Yang et al., 2022). Another study (Gholipour et al., 2022) reported the effect of high-intensity interval training on AD. The results showed that high-intensity interval training can reduce hippocampal oxidative stress and Aβ deposition, reduce neuronal damage, and improve AD symptoms. Based on the above information, exercise training can be used as a potentially effective strategy to improve the activity of antioxidant enzymes in neurons, reduce the release of mitochondrial reactive oxygen species and levels of oxidative stress and neuronal apoptosis, and ultimately delay the progression of AD.

### Strength and limitations

Our study has several strengths. To our best knowledge, this study is the first bibliometrics analysis to evaluate hotspots and frontier in the field of exercise and AD research. Publications were searched from the SCI-E of Web of Science. A total of 396 scholarly journals with 1,104 publications on exercise and AD research were used in our study. This research included the analysis of the number of publications, citations, h-index, subject categories of Web of Science, collaboration analysis among countries/institutions, co-citation analysis of references/authors, and analysis of keywords.

This study still has some limitations. First, we only searched the literature in the SCI-E of the Web of Science Core Collection database, since different databases have different properties, such as citation counting and export formats. Second, English papers accounted for 98% of the included papers in our study, because Web of Science database mainly indexed papers written in English. This may lead us to ignore relevant research published in other languages. Third, some recent publications of high quality may not received enough attention because of low citation frequency, whilst older articles have accumulated more citations. This may undermine the significance of more recently-published articles. Therefore, readers should be aware that all these may lead to bias for our results.

## Conclusion

This study collected relevant literature on exercise and AD, analyzed information of major countries/regions, institutions, and core journals in this field, and summarized research hotspots and frontiers. The number of publications on exercise and AD has been increasing rapidly, especially in the past 10 years. Most of these publications are associated with neurosciences, geriatrics, and gerontology, but they also involve sports science, psychology, behavioral science, and rehabilitation. From this perspective, enhanced inter-agency and interdisciplinary cooperation is essential for the progress and development of this scientific field. The countries in America and Europe, especially the United States, dominate in terms of publication and research collaboration on exercise rehabilitation of AD. Asian countries need to actively seek international cooperation to enhance their global influence for the further development of this field. “Amyloid beta,” “oxidative stress,” and “exercise program” are considered the current research hotspots and frontiers in this field. Although the included studies contribute to the understanding of the underlying pathways of exercise on AD, the mechanism remains unclear. In addition, no study compared the different effects of various intensities, frequencies, and durations on the related mechanism. Further studies evaluating differences in exercise programs are necessary.

## Author contributions

JG and BC contributed to conception and design of the study and revised the manuscript. YF, BC, and WZ collected and analyses the data. BC, JG, YF, and GS wrote the manuscript. All authors read and approved the final version of manuscript.
